# Influence of shear stress and size on viability of endothelial cells exposed to gold nanoparticles

**DOI:** 10.1007/s11051-017-3993-5

**Published:** 2017-09-11

**Authors:** C. Fede, Giovanna Albertin, L. Petrelli, R. De Caro, I. Fortunati, V. Weber, Camilla Ferrante

**Affiliations:** 1 Dipartimento di Neuroscienze-Istituto di Anatomia, Università di Padova, Via Gabelli 65, 35131 Padova, Italy; 2 Dipartimento di Scienze Chimiche e UdR INSTM, Università di Padova, Via Marzolo 1, 35131 Padova, Italy

**Keywords:** Nanotoxicity, Endothelial cells, Gold nanoparticles, Microfluidic, Fluorescence microscopy, Nanobiomedicine

## Abstract

Screening nanoparticle toxicity directly on cell culture can be a fast and cheap technique. Nevertheless, to obtain results in accordance with those observed in live animals, the conditions in which cells are cultivated should resemble the one encountered in live systems. Microfluidic devices offer the possibility to satisfy this requirement, in particular with endothelial cell lines, because they are capable to reproduce the flowing media and shear stress experienced by these cell lines in vivo. In this work, we exploit a microfluidic device to observe how human umbilical vein endothelial cells (HUVEC) viability changes when subject to a continuous flow of culture medium, in which spherical citrate-stabilized gold nanoparticles of different sizes and at varying doses are investigated. For comparison, the same experiments are also run in multiwells where the cells do not experience the shear stress induced by the flowing medium. We discuss the results considering the influence of mode of exposure and nanoparticle size (24 and 13 nm). We observed that gold nanoparticles show a lower toxicity under flow conditions with respect to static and the HUVEC viability decreases as the nanoparticle surface area per unit volume increases, regardless of size.

**Electronic supplementary material:**

The online version of this article (10.1007/s11051-017-3993-5) contains supplementary material, which is available to authorized users.

## Introduction

The widespread use of nanoparticles (NP) in commercial products has prompted awareness and concern towards their impact on human health and environmental safety (Cuddy et al. 2016; Feliu and Fadeel 2010; Vance et al. 2015). As a consequence, research work in the field of “nanotoxicity” has seen a steady increase with dedicated journals or journal sections. The better results in this field can be achieved by testing NP hazardous effects directly on live animals, although strict ethical guidelines must be applied to avoid their indiscriminate use (Almeida et al. 2011; Dykman and Khlebtsov 2012). For this reason, the large majority of tests are carried out on cell lines in vitro. These experiments allow determining NP toxicity and uptake mechanisms in single cell lines, as well as other specific cell responses like, for example, reactive oxygen species (ROS) production or specific protein expression. The major drawback of this approach is the discrepancy of the uptake and viability data recorded in vitro with the one observed in more organized systems such as tissues or organs, or finally in live animals (Khlebtsov and Dykman 2011; Mahmoudi et al. 2012). This discrepancy can be in principle avoided if the conditions in which the in vitro experiments are carried out resemble the one experienced by the same cell type in the in vivo organs in which they reside. On this front, microfluidic devices (MFD) offer a very promising and powerful tool, since it is possible to engineer their structure to satisfy the previous requirements (Mahto et al. 2015). There are already many examples of MFD, in which one or more cell lines are grown in co-culture to simulate the in vivo organ functions (Sakolish et al. 2016). Among these prototypes, there are devices simulating the alveolar-capillary interface of human lungs (Huh et al. 2010), the intestinal epithelium and liver tissue (Mahler et al. 2009), and the blood-brain barrier (Wang et al. 2016).

One of the main features of MF is the possibility to grow cells under continuous flow, and therefore experiencing a shear stress whose magnitude can be varied by simply changing the volumetric flow rate. For this reason, MFD are ideally suited to simulate the environment experienced by those cells, which are subject to shear stress induced by body fluids. As an example, endothelial cells grown in MFD under a constant flow display an elongated shape along the flow direction (Song et al. 2005; Young and Simmons 2010) similar to their shape in live blood vessels. Therefore, MFD supply a powerful tool to study the reaction of this kind of cells to external agents, which are either intentionally injected or end up in the blood flow.

To our knowledge, the first work exploiting MFD investigated the interaction of pegylated poly(lactic acid) micro- and nanoparticles with epithelial PC3 and LNCaP cells which expressed or not prostate-specific membrane antigen (PSMA) (Farokhzad et al. 2005). The authors observed that under static conditions or at low flow rates (shear stress <1 dyn cm^−2^), the particles will bind to the cells, whereas at high flow rates (shear stress ~ 5 dyn cm^−2^), the particles did not adhere on the cell surface.

In the last 5 years, the use of MFD has been applied to different cell lines and NP types looking at the influence of shear stress on parameters, such as, cell viability, NP uptake, and ROS production (Kim et al. 2011; Mahto et al. 2010; Sambale et al. 2015; Samuel et al. 2012; Yang et al. 2013). All these works show that cell response to NP changes from static to flow exposure and is often influenced by the shear stress experienced by the cells. For example, semiconductor quantum dots toxicity towards fibroblast cells is lower when administered under flow conditions (Mahto et al. 2010), whereas mesoporous silica nanoparticles appear to be more toxic for endothelial cells under flow conditions (Kim et al. 2011).

Freese et al. (2014) investigate SiO_2_ NP uptake into human umbilical vein endothelial cells (HUVEC) cultivated under cyclic stretch conditions. Stretch (5%) was applied to HUVEC grown on a flexible silicon membrane subject to cyclic elongation with a 1Hz frequency and exposed to a growth medium for 48 h. Different sizes and surface modifications of SiO_2_ NP surface (−COOH, −NH_2_, and –OH groups) were investigated. The results show that, for all kinds of NP, cytotoxicity does not change significantly when HUVEC are grown under stretch or static conditions, whereas NP uptake is always lower when the experiments are carried out under stretch conditions. Still, looking at the results, there are slight differences between different kinds of NP, for example, 30 nm NP seem to be internalized more than 70 nm ones, and among the 70 nm NP, the ones with –COOH end groups seem to be internalized more than both the ones with –OH and –NH_2_ end groups.

Recently, Klingberg et al. (2015a) applied MFD technology to observe the interaction of gold NP (Au NP) with HUVEC under flow conditions. They used 80 nm commercial Au NP unmodified or surface modified with antibodies against the intracellular adhesion molecule 1 (ICAM-1). The average hydrodynamic diameter of the NP increased when mixed to the cell culture medium, sign of either adsorption of serum proteins or NP aggregation. HUVEC were cultivated under static or flow conditions for 24 h. Afterwards, Au NP were administered for 3 h, using alternatively static or flow conditions. Overall, four types of samples were investigated as follows: 24 h static growth + 3 h static exposure, 24 h static growth + 3 h flow exposure, 24 h flow growth + 3 h static exposure, and 24 h flow growth + 3 h flow exposure. The uptake of unmodified Au NP is lower for cells grown under flow conditions and when NP are administered under flow, whereas the higher uptake is present when both growth and administration are carried out under static conditions. An increase in NP uptake by cells grown under flow conditions with respect to static ones was observed only for anti-ICAM-1-modified Au NP when HUVEC are activated with tumor necrosis factor.

All these works show that mechanical forces play an important role in the response of cells to NP, but still the possible combination of this parameter with other NP characteristics such as dimensions, chemical composition, and surface properties (average zeta potential, specific end groups, etc.) can modify the overall toxicity or NP uptake mechanism by cells.

Recently, we compared the change in viability of HUVEC exposed for 24 h with varying doses of citrate-capped Au NP (13 nm diameter) under flow vs. static conditions (Fede et al. 2015). HUVEC were grown inside multiwells (static exposure) or inside a linear MFD (flow exposure). For Au NP concentrations higher than 5 × 10^10^ NP ml^−1^, we observed a marked decrease in viability for cells grown under static conditions in comparison with the one grown under flow conditions. Moreover, Au NP uptake, estimated through inductively coupled plasma-atomic emission spectroscopy (ICP-AES) experiments, is one order of magnitude larger under static vs. flow exposure. Starting from these results, we decided to test the influence that the combination of a change in NP size and the application of shear stress can have on the viability of HUVEC. In order to avoid drastic changes in the uptake mechanism of the NP, we just doubled the NP size (24 nm), while we did not change the capping agent (citrate) or the experimental conditions for static and flow exposure.

## Materials and methods

### Synthesis and characterization of Au NP

Gold(III) chloride solution of 30% in dilute HCl and sodium citrate dihydrate were both purchased from Sigma-Aldrich and used without further purification. Gold colloidal dispersion was prepared according to Turkevich procedure (Turkevich et al. 1951).

UV-Vis spectra of Au NP solutions were recorded with a Varian Cary 5 spectrometer. Transmission electron microscopy (TEM) in bright-field and high-resolution modes was employed to assess Au NP morphology. A field-emission gun (FEG) Tecnai F20 Super-twin (S)TEM working at 200 keV was used. Dynamic light-scattering (DLS) measurements were performed with a Malvern Zetasizer Nano ZS. Fluorescence correlation spectroscopy (FCS), fluorescence imaging, and fluorescence lifetime imaging microscopy (FLIM) were all carried out on an Olympus FV300 laser scanning confocal microscope exploiting a two-photon absorption process for the excitation of Au NP. Excitation was provided by a Ti:Sapphire fs Laser system (820 nm, 76 MHz; Coherent, Mira900-F), focused onto the sample using a 60 × water immersion microscope objective. A photomultiplier tube was used for fluorescence imaging, whereas for FCS and FLIM, PicoHarp 300 electronics (PicoQuant) for single-photon counting and two avalanche photodiodes (SPAD, MPD, Italy) were used. Details on these techniques and the physical observation that they allow measuring are given in Electronic supplementary materials (ESM) 1 and 2.

### Preparation of microfluidic devices and flow experiments

Simple MFD were built with polydimethylsiloxane (PDMS, Sylgard 184, Dow Corning, MI, USA), employing the replica molding technique as previously described (Carlotto et al. 2011; Rossetto and Ferrante 2014).

To test NP stability in a solution under flow conditions, a Y-shaped MFD with two separate inlets and one outlet was employed. The two inlet channels were 57 μm wide, whereas the main channel was 87 μm wide and 1 cm long. The channel depth was approximately 32 μm. Au NP in water were injected in one inlet, whereas in the other one, a specific fluid was injected to be slowly mixed with the NP solution, as a consequence of the laminar flow typical of MFD. The chosen fluids were pure water, serum at 2% in water, and complete culture medium with 2% serum. A single syringe pump (KdScientific KDS 210) pushing two syringes injected the different solutions in the two inlets at the same volumetric flow rate of 1 μl min^−1^.

For cell cultures and cell viability tests under flow conditions, a linear microchannel (length 40 mm, width 2 mm, and height 150 μm) with an inlet and an outlet was used. A syringe pump (World Precision Instruments, Inc., Sarasota, FL, USA) injected the media at a constant flow rate of 5 μl min^−1^ inside the MFD.

### Cell line culture and viability tests

The human cell line HUVEC was obtained from American Type Culture Collection (ATCC, Rockville, MD, USA) and characterized by flow cytometry using anti-von Willebrand factor antibody as indicated in our previous works (Albertin et al. 2011). HUVEC were cultured in mono-layer and maintained in endothelial cell growth medium (International PBI) supplemented with 2% fetal calf serum and antibiotics (100 units ml^−1^ streptomycin and 100 units ml^−1^ penicillin G). Cells were kept at 37 °C in a humidified atmosphere containing 5% CO_2_ and used until 11 passages. The endothelial phenotype of the isolated cells was confirmed by flow cytometry using anti-von Willebrand factor antibody.

To evaluate the effect of HUVEC cell exposure to Au NP under static conditions, the cells were plated (200 cells mm^−2^ in 500 μl in 24 multiwells (MW) fibronectin pre-coated) and allowed to attach. Then, Au NP were diluted to appropriate concentrations in cell culture medium and immediately administered to the cells for 24 h. MFD were used to assess exposure under flow conditions. To this end, sterilized MFD were firstly rinsed with PBS and thereafter coated with fibronectin (1 μg cm^−2^). A HUVEC cell suspension (150–200 cells mm^−2^) was then injected through the inlet of the device and allowed to attach for 1 h at 37 °C. Afterwards HUVEC were exposed to varying concentrations of Au NP in cell medium, injected by the syringe pump at 5 μl min^−1^ for 24 h. The number of exposed cells and the level of confluence were similar to the static conditions.

Only two NP concentrations were investigated: 4.3 × 10^11^ and 1.3 × 10^12^ NP ml^−1^, selected because our previous work showed that viability decreases only above ~ 5 × 10^11^ NP ml^−1^. Higher concentrations cannot be investigated because the 1.3 × 10^12^ NP ml^−1^ solution was obtained using the mother solution at the highest feasible concentration.

The cell viability test employed to investigate NP toxicity in MW as well as in MFD was Live/Dead cell staining kit (Sigma-Aldrich®) that assesses cell viability by calcein-AM and propidium iodide (PI) fluorescence, which stain viable and dead cells, respectively. Briefly, cells were seeded on a glass coverslip or in a MFD and allowed to attach. After incubation with Au NP (24 h), cell culture medium was removed, and the cells were washed twice in PBS. The cells were then incubated with calcein-AM 10 μM and ethidium homodimer 15 μM, in PBS, for 20 min at 37 °C and washed twice in PBS. Stained cells were then detected by fluorescence microscopy (Leica Microsystems): the calcein generated from calcein-AM by esterase in viable cells emitted green fluorescence (excitation, 490 nm; emission, 515 nm), whereas PI, intercalated with DNA by passing through disordered areas of dead cell membrane, emitted red fluorescence (excitation, 535 nm; emission, 617 nm). For every administered NP concentration, the cell survival rate was measured as the number of living cells expressed as percentage of the number of control cells, which underwent the same processing steps but did not receive NP.

### Statistical analysis

We performed a statistical analysis (*t* test) to analyze significant differences when comparing treated cells with controls, static with flow conditions (considering the same concentration of NP), and 24 with 13 nm NP (considering the same NP surface per unit volume).

## Results and discussion

Au NP characterization is a necessary step if one wishes to confront data from different experiments. To this end, it is important to gain information for NP both before and after exposure to cell culture medium, since the latter can alter the dimensions and surface properties of the NP (Favi et al. 2015; Pino et al. 2014; Yang et al. 2014).

The dimensional distribution of the Au NP metal core was recorded with high-resolution TEM imaging for the batch employed in this work (from now on called batch 24 nm**)**. The average radius was 12 nm, the radius standard deviation was 4 nm, and the average size, i.e., the average diameter, was 24 ± 8 nm. Figure S1 of the ESM shows a TEM picture and the size distribution. This distribution, together with the weighted amount of gold used to synthesize the NP, are the data used to quantify NP concentration in solution. In particular, as previously described by us, the whole distribution of sizes was used to determine the number of NP per milliliter as well as the NP molar concentration (Fede et al. 2015). A summary of the concentrations of Au NP employed in this work expressed with different units of measurements is given in Table 1. This table is provided to facilitate comparison with literature data, since the concentrations of colloidal solutions were often reported with only one of the proposed scales. Table 1 also reported the same data for the second batch of Au NP (batch 13 nm) previously investigated by us and characterized by smaller dimensions (average diameter 13 nm ± 3 nm) (Fede et al. 2015).

**Table 1:** Au NP concentrations calculated by the distribution of TEM diameters for batch 24 nm and batch 13 nm

Number (NP ml^−1^)	Surface area per volume (cm^2^ ml^−1^)	NP concentration (M)	Au concentration^a^ (M)	Au concentration^a^ (mg ml^−1^)
Batch 24 nm				
(1.3 ± 0.2) × 10^12^	23.0 ± 5.5	(2.1 ± 0.3) × 10^−9^	1.0 × 10^−3^	2.0 × 10^−1^
(4.3 ± 0.5) × 10^11^	7.7 ± 1.8	(7.1 ± 0.9) × 10^−10^	3.4 × 10^−4^	6.6 × 10^−2^
Batch 13 nm^b^				
(2.5 ± 0.1) × 10^12^	16.1 ± 1.2	(4.2 ± 0.5) × 10^−9^	4.1 × 10^−4^	8.0 × 10^−2^
(1.0 ± 0.2) × 10^12^	6.4 ± 0.5	(1.7 ± 0.2) × 10^−9^	1.6 × 10^−4^	3.2 × 10^−2^
(5.0 ± 0.5) × 10^11^	3.2 ± 0.2	(8.5 ± 1.0) × 10^−10^	0.8 × 10^−4^	1.6 × 10^−2^

^a^Data are affected by a 7% error

^b^Data from Fede et al. (2015)

In the solution, the actual size of the Au NP can be different from the one observed with TEM because of the solvation shell. DLS and two-photon FCS experiments can be used to assess the hydrodynamic diameter of the nanoparticle in pure water. Under two-photon excitation at 820 nm, Au NP exhibited a weak fluorescence mainly assigned to a radiative relaxation of surface defects. The defects number increased with the excitation laser power, as a consequence of the movement of the capping agent molecules at the interface between the NP and the solvent (Fortunati et al. 2014; Loumaigne et al. 2010). DLS and FCS, carried out on Au NP of batch 24 nm in water, gave equal results with an estimate of the hydrodynamic diameter of 30 ± 10 and 30 ± 4 nm, respectively. This diameter is slightly larger than the one observed by TEM because it accounts also for the citrate shell capping the NP. NP from batch 13 nm displayed a hydrodynamic diameter of 18.6 ± 2.4 nm.

Exposure of Au NP to biological media can change both the average dimensions of the NP, because of protein adsorption on the NP surface (Pino et al. 2014), and the tendency to aggregate (Yang et al. 2014), because of the high ionic strength of biological media.

**Fig. 1:**
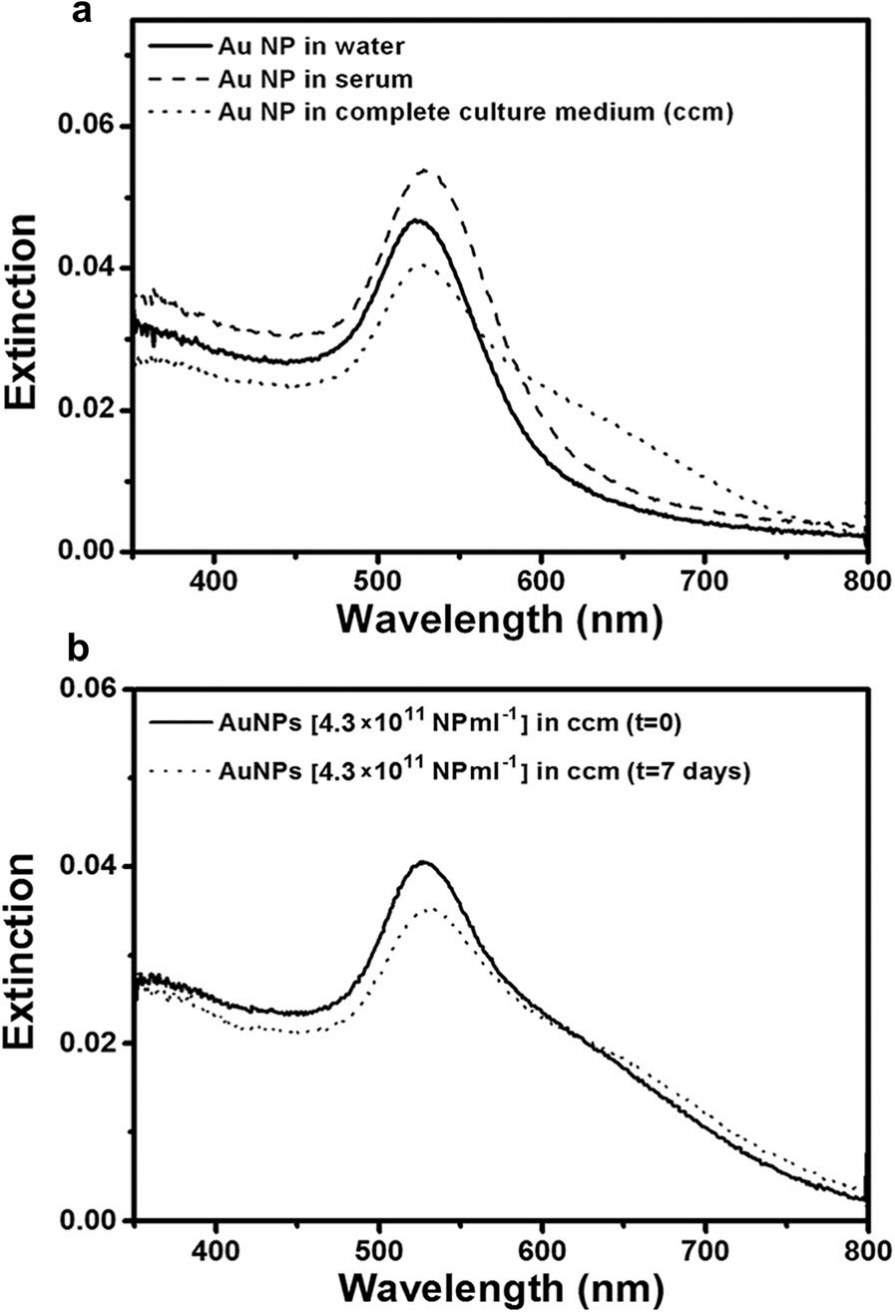
UV-Vis spectra of Au NP solutions **a** in different media (4.3 × 10^11^ NP ml^−1^) and **b** after a few minutes or after 1 week in complete cell culture medium (with 2% serum)

In order to observe if there were changes in NP behavior, the UV-Vis absorption spectra of batch 24 nm (concentration, 4.3 × 10^11^ NP ml^−1^) were measured in different media and as a function of exposure time. The results are depicted in Fig. 1a, b. In pure water (black line in Fig. 1a), our sample showed the typical absorption spectrum of well-dispersed Au NP with the characteristic plasmonic resonance band at 525 nm. In serum (dashed line in Fig. 1a), the absorption showed a slight shift of 5 nm sign that the NP were still well dispersed and with similar dimensions. In the complete culture medium (with 2% serum), the absorption spectrum of the NP (dotted line in Fig. 1a) still maintained the main absorption band at 527 nm, together with a pronounced shoulder in the red region. These spectral features suggest that in the whole culture medium, there were both mono-dispersed Au NP and small-medium size aggregates. Given the high ionic strength of the culture medium, stability of single Au NP or aggregates in the solution must be mediated by changes in the NP surface properties. Indeed, it is well known that citrate-capped Au NP, when exposed to serum proteins, develop a protein corona on the surface usually formed by a single protein layer (Kohli et al. 2013; Treuel and Nienhaus 2012). Another important information concerns stability of the Au NP dispersion in the cell culture medium over time. The pictures in Fig. 1b show the absorption spectra recorded for Au NP 4.3 × 10^11^ NP ml^−1^ in complete cell culture medium after a few minutes and after 1 week. The spectra are almost identical, with a slight decrease of intensity for the sample after 1 week, a sign that the solution was very stable also for this long amount of time. Our previous studies on citrate-capped Au NP of smaller size (batch 13 nm) confirmed this behavior. FCS experiments run on batch 13 nm confirmed an increase in the NP diameter compatible with the deposition of a protein layer on the NP surface as well as the presence of NP aggregates in solution (Fede et al. 2015).

**Fig. 2:**
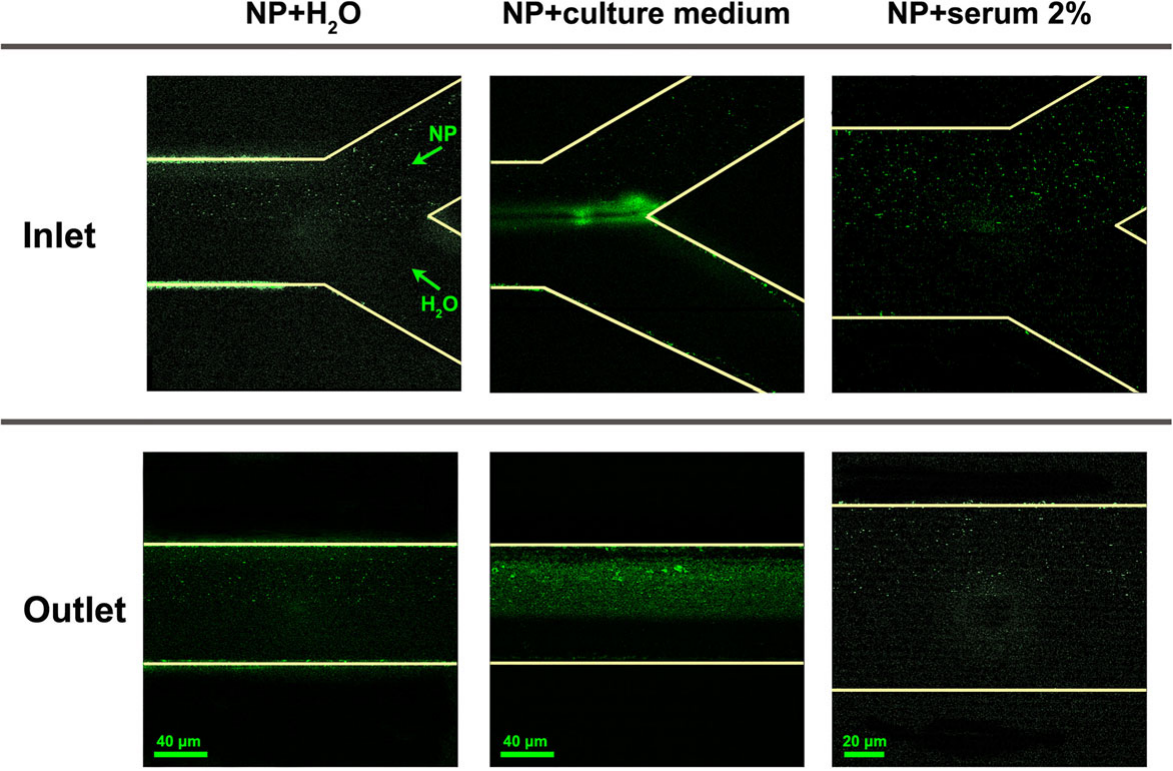
Two-photon excited fluorescence images of the inlets (*top*) and of the main channel towards the outlet (*bottom*) of a Y-shaped MFD in which a solution of Au NP in water is mixed with pure water (*left column*), complete culture medium with 2% serum (*central column*) or 2% serum in water (*right column*). The Au NP solution is always injected in the upper inlet of the Y. Fluorescent emission/scattering form the NP or NP aggregates is rendered in *green*

So far, the behavior of Au NP has been observed in macroscopic samples under static conditions. Since the experiments were also carried out under flow conditions, we used a Y-shaped MFD to slowly mix Au NP in water with alternatively pure water, serum, and complete culture medium (2% serum). Images of the fluorescence emitted by the Au NP, after two-photon excitation at 820 nm, were taken with the scanning confocal fluorescence microscope adjusting the depth position, so as to gather the signal only from the channel center. The pictures in Fig. 2 show fluorescence images for Au NP solutions at the entrance of the Y-shaped microfluidic device (upper raw) and towards the outlet (bottom raw). Au NP in water were always injected on the upper inlet, whereas the other medium was injected from the lower one. The left column shows pictures of Au NP mixed with pure water, and only some small fluorescence speckles were visible in the upper part of the channel due to the weekly fluorescent NP. The two pictures in the central column display the mixing of the Au NP with the complete culture medium. The bright-green speckles clearly visible in the upper part of the channel both at the inlet and towards the outlet were signs of aggregation for Au NP. The signal gathered by the microscope was likely a mixture of Au NP fluorescence and scattered laser light (in particular for large NP aggregates). Finally, when serum, i.e., protein solution, was injected with Au NP, right column pictures, still few speckles of fluorescence were observed, sign that NP were mono-disperse or only very small aggregates were present. The increase in brightness observed for the NP in comparison with pure water can be a consequence of NP surface modification because of protein adsorption (Shang et al. 2012), although we cannot completely exclude that it is also promoted by scattering from small aggregates.

These tests in the MFD, together with the UV-Visible analysis in static mixing condition, showed that Au NP behavior when exposed to serum or to the complete culture medium did not change substantially from static to flow conditions.

Figure 2 also shows that some NP stick on the sidewalls of the MFD; in principle, this can alter the concentration of NP flowing inside the MFD. We observed that NP adsorption occurred only for the first few minutes of infusion, and afterwards, the brightness on the channel walls did not change. We estimated that changes in NP concentration should occur only in this short time interval which was negligible when compared with the 24 h time span used in the viability tests performed on cell cultures and described in the following.

**Fig. 3:**
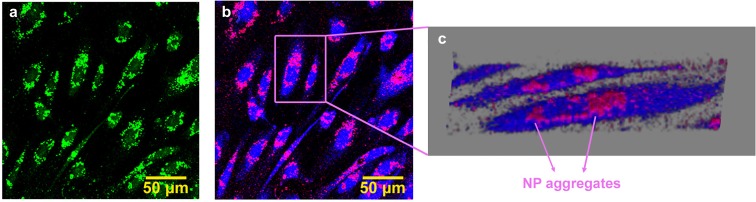
Two-photon fluorescence confocal microscope image (**a**) and two-exponential fit FLIM image (**b**) of HUVEC cells incubated with Au NP 1.3 × 10^12^ NP ml^−1^ for 24 h (static conditions), collected at 3 μm from the coverglass; 3D reconstruction of a stack of 11 FLIM images (**c**). In FLIM images, the red signal is the amplitude of short lifetime contribution (assigned to Au NP emission) and the blue signal is the amplitude of long fluorescence lifetime term (assigned to residual cell autofluorescence)

A qualitative estimate of Au NP uptake by HUVEC was accomplished through optical microscopy investigations. The pictures in Fig. 3 show two-photon fluorescence images, recorded with the scanning confocal microscope, of HUVEC grown in MW after exposure to a solution of Au NP (1.3 × 10^12^ NP ml^−1^) in cell culture medium for 24 h. The sample was two photons excited at 820 nm, and the light emitted by the sample was suitably filtered, so as to minimize scattered light. The emitted light intensity was rendered in green (Fig. 3a), and it suggested that a large number of NP aggregates were either deposited on cells or internalized, although also cell autofluorescence can still contribute to the image. To get a better distinction between NP fluorescence/scattering and cell autofluorescence and gather a better insight in the real uptake of NP inside the cell, FLIM experiments were also carried out. Details on the experimental set-up and the data analysis are given in ESM 1. Pictures 3B and 3C show FLIM images of the same sample depicted in picture 3A. The FLIM image reports the amplitude map of the long fluorescence lifetime component (1.8 ns) in blue and the amplitude of the short component (≈ 200 ps) in red. The longer component was associated to cell autofluorescence, and it was calibrated by FLIM analysis of a control HUVEC sample without NP. The shorter one was instead linked to the fast response of Au NP. Picture 3C is a 3D-FLIM image obtained from the 3D reconstruction of FLIM images, collected at different distances from the coverglass (steps of 1 μm). Both images clearly show that Au NP form aggregates, which are mainly deposited directly on the coverglass, where no cell is present, and on the outside surface of the cells. Indeed, this picture shows that a large amount of NP form aggregates, sticking on the outer cell membrane, and only few NP can be localized inside the cytoplasm (see also the movie in ESM 2). Fluorescence and FLIM maps were also recorded for the 4.3 × 10^11^ NP ml^−1^ and these pictures are included as Fig. S4 in the ESM 1.

**Fig. 4:**
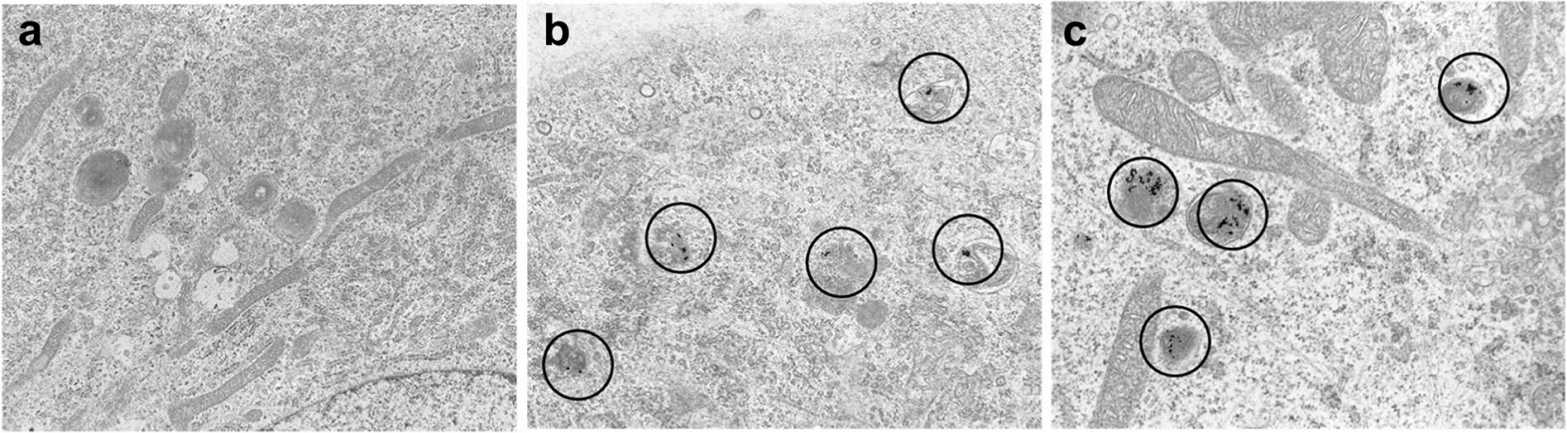
TEM images for HUVEC grown in multiwells: **a**, **b** 15,000 × and **c** 20,000 ×. **a** Control cells, 24 h; **b** 5 h incubation Au NP 4.3 × 10^11^ ml^−1^, batch 24 nm; **c** 5 h incubation Au NP 5.0 × 10^11^ ml^−1^, batch 13 nm

A clear confirmation that NP were indeed internalized inside the cell was provided by TEM analysis (details in ESM 1). TEM pictures are given in Fig. 4 and show that the NP internalized by endocytosis are localized in the cytoplasmic endosomes, as in the case of batch 13 nm.

Internalization of citrate-capped gold NP with similar dimensions by endothelial cells has been already reported in the literature (Freese et al. 2012b; Klingberg et al. 2015b). A thorough investigation on internalization observed that uptake occurred through clatrin-mediated endocytosis and aggregates of few Au NP were localized in endosomes or lysosomes inside the cytosol, in agreement with our data (Klingberg et al. 2015b). It is important to point out that a recent article suggested that the internalization pathway could affect cell viability (Guarnieri et al. 2014).

**Fig. 5:**
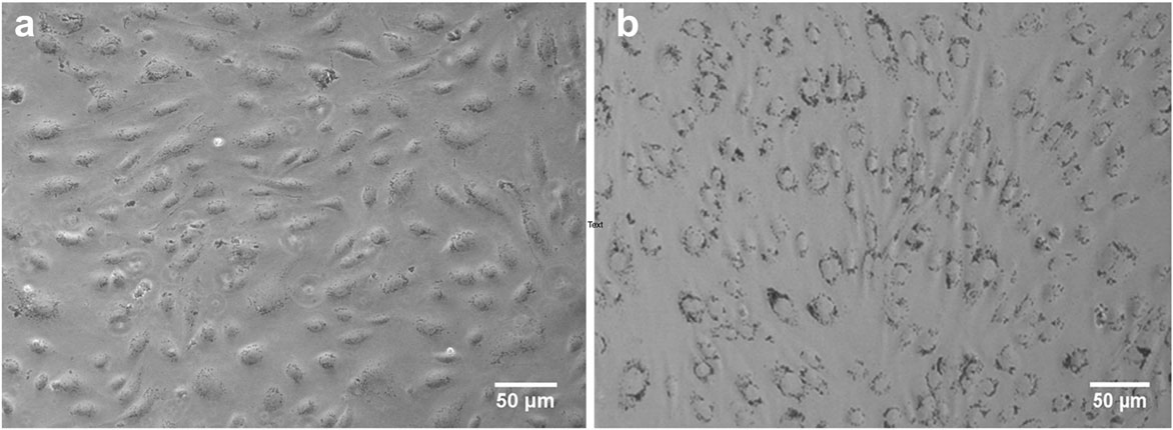
Bright-field images. HUVEC cells treated for 24 h with Au NP (1.3 × 10^12^ NP ml^−1^; batch 24 nm) in flow conditions in a MFD (**a**) or in static conditions in MW (**b**)

The pictures in Fig. 5a, b, recorded in reflection mode with an optical microscope (Leica Microsystem), portray HUVEC exposed for 24 h to NP (1.3 × 10^12^ NP ml^−1^) inside the MFD and in MW, respectively. These pictures show that under static exposure, i.e., in MW, large Au NP aggregates stick on the HUVEC cells, whereas under flow conditions, i.e., in MFD, fewer NP were attached to the cell. Similar results were also reported for batch 13 nm as confirmed also by the lower Au NP content measured by ICP-AES showing that the percentage of gold accumulation was very low (0.17% with respect to the total provided amount) in flow compared with the culture in well (29.2%) (Fede et al. 2015). Furthermore, while in MFD, HUVEC still had the elongated form typical of this cell type, in MW they had a more rounded shape: this is signal of cell stress, as indicated by previous reports (Ruoslahti 1997) and as reported by a work of Marx et al. (2001), which observed how changes in the cytoskeleton occur when endothelial cells respond to chemicals that alter cell properties. As already discussed in our previous work, to understand the differences observed in the number of Au NP that were deposited on cell membrane under flow vs. static conditions, one should take into account processes like sedimentation, diffusion, and advection (the latter only in flow conditions) together with specific interaction forces causing adhesion of NP on cell surface and the effect of shear stress when flow was applied (Decuzzi et al. 2005; Hinderliter et al. 2010).

A Live/Dead assay was used to test cell viability in both MW and MFD of HUVEC exposed to two different concentrations of Au NP for 24 h. In MFD, the applied flow rate was 5 μl min^−1^ corresponding to a shear stress of 0.1 dyn cm^−2^. Even if the applied shear stress was low in comparison with values found in many physiological processes, it was anyway similar to some in vivo conditions, such as, for example, in bifurcation of vessels (Malek et al. 1999). We selected Live/Dead test because it allowed performing the analysis directly on the coverglass of the device, without removing and counting the cells, and reducing count errors (Fede et al. 2014). The results are summarized in Table 2: only at the highest concentration was there a marked reduction of viability in MW (39.0 ± 0.2%) as well as in MFD (75.0 ± 3.7%), although the reduction was sensibly larger in MW with respect to MFD. In fact, the statistical analysis confirmed that there was a significant difference (*p* < 0.01, *t* test) between static and flow conditions at the concentration of 1 × 10^12^ NP ml^−1^.

**Table 2:** Cell viability in multiwells (MW) and in microfluidic device (MFD) after 24 h of exposure with batch 24 nm and batch 13 nm NP

Au NP ml^−1^	MW	MFD
	Cell viability (batch 24 nm)	
	(%)	(%)
(4.3 ± 0.5) × 10^11^	89.0 ± 0.4	93.2 ± 4.7
(1.3 ± 0.2) × 10^12^	39.0 ± 0.2	75.0 ± 3.7
	Cell viability (batch 13 nm)	
	(%)	(%)
(5 ± 0.5) × 10^11^	67.7 ± 0.3	97.0 ± 4.8
(1.0 ± 0.2) × 10^12^	70.1 ± 5.9	91.2 ± 4.6

At the lower concentration, instead, a slight decrease in viability was observed in both MW and MFD. We were not able to confirm this trend with higher NP concentrations, because of the limit imposed by the native Au NP solution. Our previous experiments carried out on batch 13 nm under the same conditions confirmed this trend, i.e.: HUVEC exposed to Au NP under flow conditions in MFD displayed an overall higher viability with respect to static exposure conditions in MW (*p* < 0.01 at all the NP doses analyzed).

**Fig. 6:**
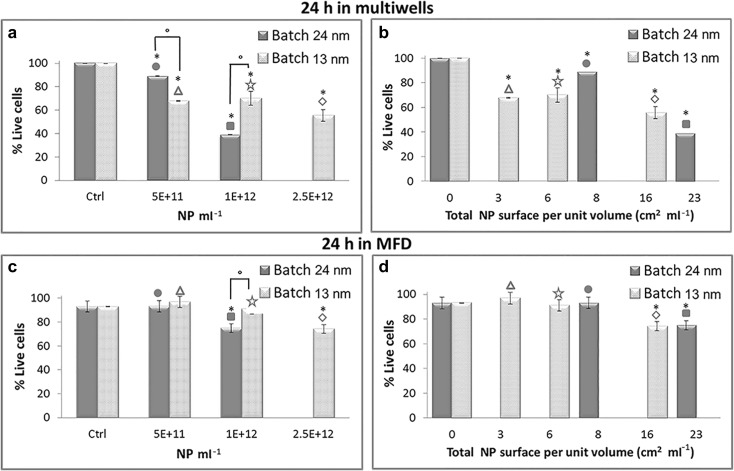
Percentage of live cells in multiwells (**a**, **b**) or in MFD (**c**, **d**) after 24 h exposure to increasing doses of Au NP, assessed by Live/Dead assay. The applied flow rate in the MFD is 5 μl min^−1^. The data are plotted against NP concentration expressed as number of NP per milliliter (**a**, **c**) or against the total NP surface area per unit volume of solution (cm^2^ ml^−1^) (**b**, **d**). Data represent mean ± standard deviation (2 ≤ *n* ≤ 4; *n* is the number of experiments with at least 150 cells counted for each sample). *Symbols on the top column* help identify the different NP solutions as reported in Table 1. *p < 0.01, *t* test treated vs. control cells; °*p* < 0.01 *t* test batch 24 nm vs. batch 13 nm. *p* < 0.01 (*t* test) also between 3 cm^2^ ml^−1^ multiwells and MFD (^∆^); *p* < 0.01 (*t* test) also between 6 cm^2^ ml^−1^ multiwells and MFD (^☆^); *p* < 0.01 (*t* test) also between 16 cm^2^ ml^−1^ multiwells and MFD (^◊^); *p* < 0.01 (*t* test) also between 23 cm^2^ ml^−1^ multiwells and MFD (^■^)

Another important parameter that can affect NP toxicity is dimensions. Indeed, this parameter is of pivotal importance to understand how Au NP interact or react with other systems, for example, when they are used in chemistry as catalyst (Hvolbæk et al. 2007; Min and Friend 2007) or in nanomedicine for photodynamic therapy (Zakaria et al. 2016; Zharov et al. 2003). In our previous work, we used smaller Au NP (batch 13 nm) with an average diameter of 13 nm. Characterization of batch 13 nm NP was thoroughly described in reference (Fede et al. 2015), and the three concentrations of batch 13 nm NP used for comparison with the present data are summarized in Table 1. The two lower concentrations were almost equal to those used with batch 24 nm, whereas the highest could not be reproduced with batch 24 nm. Also for batch 13 nm, NP viability tests were carried out under static (MW) and flow (MFD) conditions with the same Live/Dead assay. The results of these tests are summarized in Table 2 and the histograms of Fig. 6. In histograms 6a and 6c, viability is plotted against NP concentration expressed as number of NP per milliliter. The upper panel displays data for static exposure, whereas the lower one for flowing exposure. Both in MW and MFD, it appeared that at the dose 1 × 10^12^ NP ml^−1^, the larger NP (batch 24 nm) were more toxic than the smaller ones (batch 13 nm) (*p* < 0.01, *t* test). At the lower dose 5 × 10^11^ NP ml^−1^, instead it seemed that the larger NP were less toxic in MW (*p* < 0.01), whereas in MFD, both kinds of NP seemed to be non-toxic (no significant differences checked by the statistical analysis). Our experiments do not show a clear trend as a function of native NP dimensions.

The choice to express Au NP toxicity as a function of NP concentration (see Table 1) was suggested by the article of Fratoddi et al. (2015a). Comparing viability data for HeLa cells exposed to Au NP differing per size and capping agent, the authors noticed that the best trends are observed plotting viability against the number of NP per unit volume.

Many studies carried out under static exposure showed that Au NP did not appear to be toxic at very high concentrations up to 250 μM (gold atoms) (Connor et al. 2005; Fratoddi et al. 2015b; Murphy et al. 2008). Limiting the comparison with data investigating endothelial cells and spherical Au NP, it is clear that only at very high doses, of the order of 1 × 10^10^–1 × 10^12^ NP ml^−1^, citrate-capped Au NP start to affect cell viability (Freese et al. 2012b; Klingberg et al. 2015b; Ucciferri et al. 2014). In these works, NP uptake turned out to be less than 5% of the applied dose (Freese et al. 2012b) and it increased as the concentration of the applied dose was raised (Klingberg et al. 2015b). Only Sultana et al. (2015) observed that citrate-capped 15 nm NP give rise to a 50% decrease in viability already at a concentration of 2 × 10^10^ NP ml^−1^. Similar behavior, i.e., toxicity induced only at very high doses, was also observed for Au NP substituted with different capping agents (Bartczak et al. 2015; Freese et al. 2012a; Kaur et al. 2013). Furthermore, looking at these data, there was no clear trend when one compares NP of different sizes.

Another NP characteristic which can play a strategic role for the way in which NP affect cell viability was the total NP surface area per unit volume of solution (cm^2^ ml^−1^) (data summarized in the second column of Table 1). Figure 6bd, reports the viability data plotted looking at this parameter. In this case for HUVEC exposed to Au NP in MW, there was a better recognizable trend, which pointed out that cell viability decreased as the total NP surface area increased (*p* < 0.01, *t* test treated vs. control cells), regardless of the change in NP size from batch 24 nm to batch 13 nm. The same trend was observed for HUVEC grown in MFD under flow conditions (Fig. 6d). Still, when comparing static and flow exposure, the latter turned out to be less harmful: a significant reduction in cell viability was evident only for NP surface per unit volume ≥ 16 cm^2^ ml^−1^.

Schmid and Stoeger (2016) observed that NP surface area was the more relevant dose metric for pulmonary-resistant bio-persistent spherical nanoparticles. Hirn et al. (2011) found out that accumulation of Au NP in the liver with size between 1.4 and 5 nm increased steeply with decreasing size, and there was a linear dependence with respect to global surface area of NP; for NP with sizes in the range 18–200 nm, instead, accumulation did not seem to be size dependent. Looking only at literature data for cell exposed to Au NP of different sizes (Ávalos et al. 2015; Ucciferri et al. 2014), we tried to plot viability against total NP surface area per unit volume and we could not find any clear trend connecting viability to this parameter.

In many papers, viability was also plotted as a function of weighted amount of gold per unit volume or per surface area of the multiwell (Freese et al. 2012a, b; Ucciferri et al. 2014). In our opinion, it is important to plot viability against all the possible way in which the dose of NP is administered. Unfortunately, in comparison with the data available for Au NP and other cell lines, such as HeLa cells (Fratoddi et al. 2015a), data for endothelial cells are still not enough to make a sound statistical study capable to recognize which parameters are the most influential in the definition of a clear trend for their toxicity.

Concerning data recorded under flow conditions, comparison was limited to the work of Klingberg et al. (2015a), which focused on lower NP uptake under flow compared with static exposure and did not discuss viability.

The literature data recording cell viability after NP administration comparing flow and static conditions are still too few and deal with different kind of NP and cell lines. A meaningful comparison between the literature data is not yet feasible, but it is already clear that cell response changes significantly from one exposure condition to the other.

## Conclusions

When a solution of citrate-capped Au spherical NP was administered for 24 h to HUVEC, viability was modified only at very high doses above 10^11^ NP ml^−1^. We then focused attention on two parameters that can affect viability: mode of exposure and NP size (core size 24 and 13 nm). HUVEC exposed to NP under flow conditions showed a higher viability with respect to static conditions at all the investigated doses. Comparison of viability data for the two batches against dose of NP expressed as number of NP per unit volume did not show any recognizable trend, whereas if the data were plotted against total NP surface area per unit volume, there was a clear trend in viability, which decreased as this parameter increased irrespectively of NP size. The viability was significantly different between static and flow conditions at all the doses of NP analyzed, expressed as NP surface per unit volume, except for the surface of 8 cm^2^ ml^−1^. Finally, under flow conditions, a statistically significant deviation from control is observed only for surface/volume larger than 16 cm^2^ ml^−1^.

## Electronic supplementary material

ESM 1 (PDF 1354 kb).

ESM 2 (MOV 1389 kb).

## Abbreviations

FCS: Fluorescence correlation spectroscopy
FLIM: Fluorescence lifetime imaging microscopy
HUVEC: Human umbilical vein endothelial cells
TEM: Transmission electron microscopy
MFD: Microfluidic device
