# Supplementary material for: Influence of shear stress and size on viability of endothelial cells exposed to gold nanoparticles
Source: J Nanopart Res. 2017 Sep 11;19(9):316. doi: 10.1007/s11051-017-3993-5 (PMC5594036; doi:10.1007/s11051-017-3993-5)
Supplement: Supplementary file 1 — (PDF 1354 kb). [file 11051_2017_3993_MOESM1_ESM.pdf]

## SUPPLEMENTARY MATERIAL

### High Resolution TEM of the gold nanoparticles

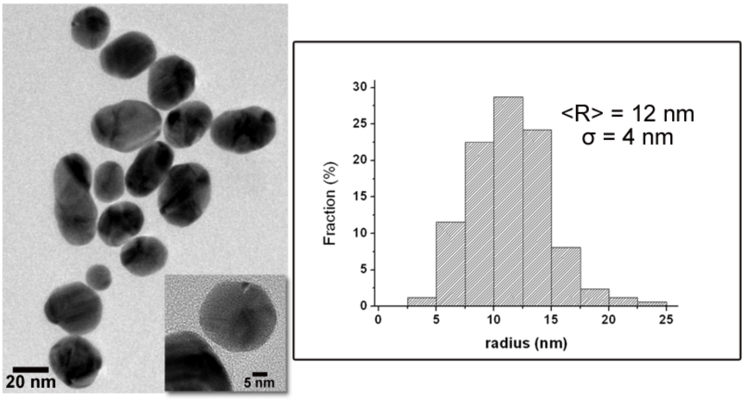


**Figure S1**: TEM image of Au NPs in bright field mode (on the left) and histogram of the size distribution (on the right) of **Batch 24 nm**.

### Determination of hydrodynamic radius by means of Fluorescence Correlation Spectroscopy

Fluorescence Correlation Spectroscopy records the fluctuations of fluorescence intensity emitted by a few fluorescent probes inside the focal volume sampled by the microscope objective in a confocal fluorescence microscope. Two independent avalanche photodiodes record simultaneously these fluctuations, and then the signals are cross-correlated to obtain the autocorrelation curve for fluorescence fluctuation, which is the FCS signal. When the fluorescent probes are subject to free-diffusion, the FCS curve is fitted using the following formula, containing both rotational and translational diffusion terms (Loumaigne et al. 2010; Loumaigne et al. 2011):

$$G_{diff}(\tau) = G_{rot}(\tau) G_{trasl}(\tau) = \left[1 + Ce^{-\tau/\tau_{ra}}\right] \left[\frac{1}{N}\left(1 + \frac{\tau}{\tau_{trasl}}\right)^{-1}\left(1 + \frac{\tau}{S^2 \tau_{trasl}}\right)^{-1/2}\right]$$

The parameters are: the rotation contrast $C$, the characteristic rotational time $\tau_{rot}$, the particle number $N$ in the focal volume, the time $\tau_{trasl}$ employed by the tracer to pass through the focal volume through a process of translational diffusion, and $S$ the focal shape-factor.

Adopting a classical hydrodynamic description, $\tau_{rot}$ and $\tau_{trasl}$ are proportional to the volume and the radius of the nanoparticle, respectively (Sauer et al. 2011):

$$\tau_{rot} = \frac{V_{NP}\eta}{K_B T} = \frac{4\pi R_h^3 \eta}{3K_B T} \qquad \tau_{trasl} = \frac{w_0^2}{8D_{trasl}} = \frac{3\pi R_h \eta w_0^2}{4K_B T}$$

where $\eta$ is the water viscosity, $K_B$ is the Boltzman constant, $T$ is the temperature, $w_0$ is the radial focal volume radius, and $R_h$ is the NP hydrodynamic radius. Assuming spherical shaped Au NPs, an estimate of the average Au NP hydrodynamic radius is calculated from extrapolated $\tau_{rot}$ values. The main advantage of using this parameter instead of $\tau_{trasl}$ for the determination of the hydrodynamic size is its independence with respect to the dimension of the focal volume.

Figure S2 shows a typical FCS curve recorded for Au NP in water.

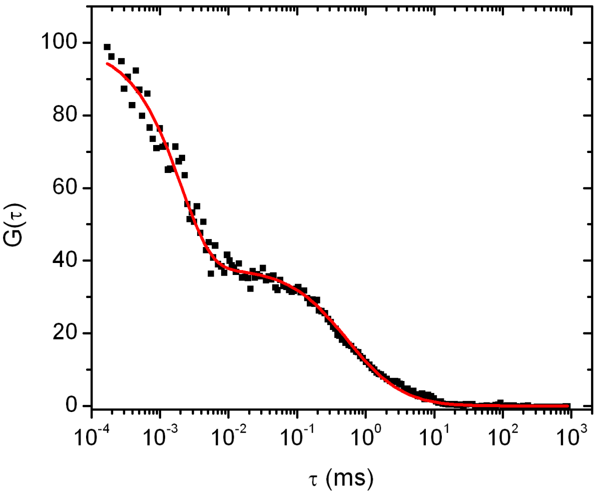


**Figure S2**: Two-photon excitation FCS curve of Au NP in water (dotted trace) and fitting curve using the formula reported above (full red line).

Loumaigne M, Richard A, Laverdant J, Nutarelli D, Debarre A. 2010. Ligand-induced anisotropy of the two-photon luminescence of spherical gold particles in solution unraveled at the single particle level. Nanoletters 10(8), 2817-2824.

Loumaigne M, Vasanthakumar P, Richard A, Débarre A. 2011. Influence of polarization and wavelength on two-photon excited luminescence of single gold nanospheres. Phys Chem Chem Phys 13, 11597-11605.

Sauer M, Hofkens J, Enderlein J. 2011. Handbook of fluorescence spectroscopy and imaging: from single molecules to emsembles. Wiley-VCH Verlag GmbH.

## FLIM: Fluorescence Lifetime Imaging Experiments

In this technique the image maps the fluorescence lifetime of the sample under investigation.

For the preparation of FLIM samples, HUVEC are seeded on a glass coverslip, incubated for 24h with different Au NPs concentrations, and fixed in formaldehyde 4% in PBS 1X. For FLIM analysis the fs laser beam (820 nm and 10 mW average power) is focused on the sample through a 60× water immersion microscope objective. The emission signal, filtered by a 750nm-shortpass and a 572/35 bandpass filters is sent to the avalanche photodiode connected to PicoHarp300 from PicoQuant. The scan area is 256×256 px. The resulted FLIM data are analyzed with the Symphotime software (PicoQuant) to generate the fluorescence lifetime map. The fluorescence decay curve from the overall map is fitted with a double-exponential model using the formula: $F(t) = A1\times\exp(-t/\tau 1)+A2\times\exp(-t/\tau 2)$, where $\tau 1$ and $\tau 2$ are the fluorescence lifetimes. Figure S3 shows the fluorescence decay curves for Au NP in water, HUVEC only and HUVEC together with Au NP.

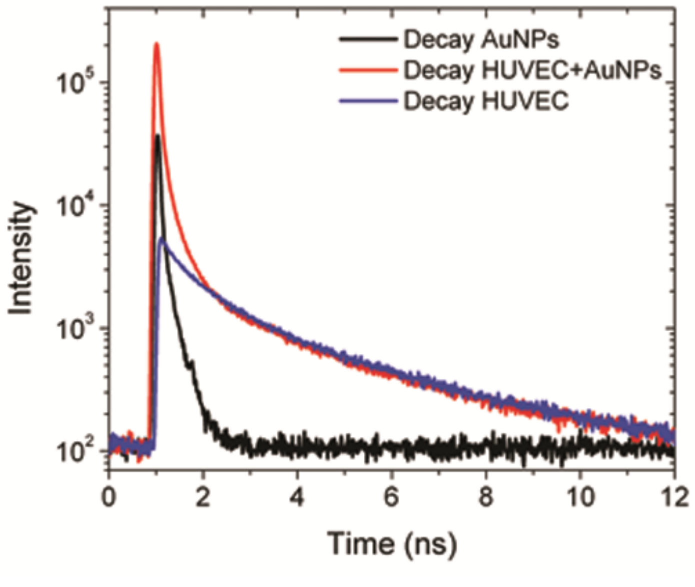


**Figure S3**: Fluorescence decay curves of Au NPs in water solution (black line), HUVEC incubated with Au NPs (red line) and HUVEC (blue line). Au NPs show a fast response, comparable to the instrument response function. This feature overlies at short times the autofluorescence nanosecond decay characterizing HUVEC.

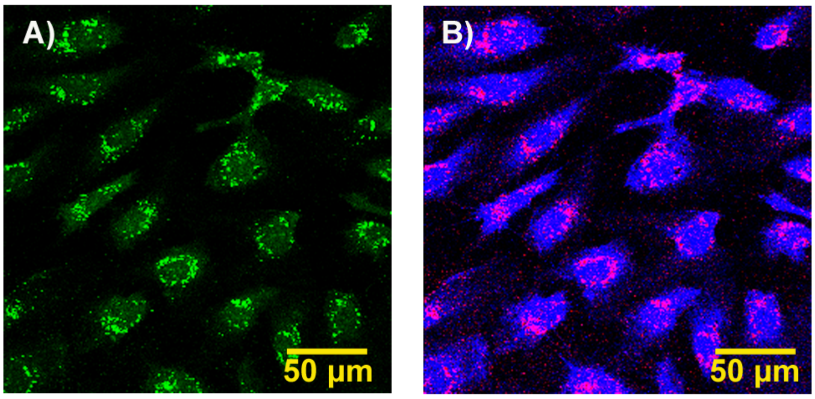


**Figure S4:** Two-photon fluorescence confocal microscope image (A) and two-exponential fit FLIM image (B) of HUVEC cells incubated with Au NP $4.3\times10^{11}$ NP $ml^{-1}$ for 24h (static conditions), collected at 3 µm from the coverglass. In FLIM image, the red signal is the amplitude of short lifetime contribution (assigned to Au NP emission) and the blue signal is the amplitude of long fluorescence lifetime term (assigned to residual cell autofluorescence).

**TEM Analysis**

HUVEC monolayers are analyzed by TEM analysis. The cells are incubated with $5\times10^{11}$ Au NP/ml, fixed in phosphate-buffered 2,5% glutaraldheyde (Serva), post-fixed in 1% osmium tetroxide ($OsO_4$) (Electron Microscopy Sciences) in 0.1M phosphate-buffered, dehydrated in a graded ethanol series, and embedded inside tissue culture dishes with Epon-812 (Electron Microscopy Sciences). Ultrathin sections (60-nm) are cut with an RMC Power-Tome ultramicrotome (Boeckeler Instruments), collected on 300-mesh grids, and stained with 1% uranyl acetate and Sato's lead. Specimens are observed in a Hitachi H-300 Electron Microscope.

**Optical Microscopy images in reflection mode**

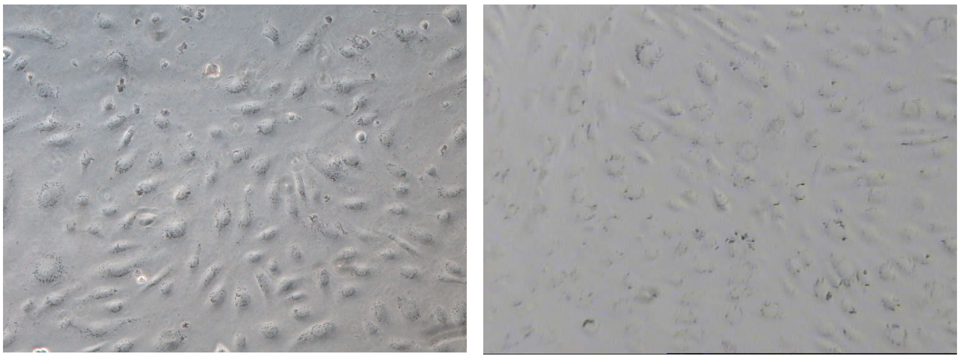

**Figure S5:** Optical microscopy images of HUVEC treated for 24 h in microfluidic device (left) and in multiwells (right) with the lower concentration of Au NP ($4.3 \times 10^{11}$ NPs/mL).
